# Implementation of an antimicrobial stewardship program on the medical-surgical service of a 100-bed community hospital

**DOI:** 10.1186/2047-2994-1-32

**Published:** 2012-10-09

**Authors:** Donald F Storey, Perry G Pate, Autumn TT Nguyen, Fung Chang

**Affiliations:** 1 Dallas ID Associates, Dallas, Texas, USA; 2 Medical City Dallas Hospital, Dallas, Texas, USA; 3 Medical Center of McKinney, McKinney, Texas, USA

**Keywords:** Antimicrobial stewardship, ASP, Small community hospital

## Abstract

**Background:**

Antimicrobial stewardship has been promoted as a key strategy for coping with the problems of antimicrobial resistance and *Clostridium difficile*. Despite the current call for stewardship in community hospitals, including smaller community hospitals, practical examples of stewardship programs are scarce in the reported literature. The purpose of the current report is to describe the implementation of an antimicrobial stewardship program on the medical-surgical service of a 100-bed community hospital employing a core strategy of post-prescriptive audit with intervention and feedback.

**Methods:**

For one hour twice weekly, an infectious diseases physician and a clinical pharmacist audited medical records of inpatients receiving systemic antimicrobial therapy and made non-binding, written recommendations that were subsequently scored for implementation. Defined daily doses (DDDs; World Health Organization Center for Drug Statistics Methodology) and acquisition costs per admission and per patient-day were calculated monthly for all administered antimicrobial agents.

**Results:**

The antimicrobial stewardship team (AST) made one or more recommendations for 313 of 367 audits during a 16-month intervention period (September 2009 – December 2010). Physicians implemented recommendation(s) from each of 234 (75%) audits, including from 85 of 115 for which discontinuation of all antimicrobial therapy was recommended. In comparison to an 8-month baseline period (January 2009 – August 2009), there was a 22% decrease in defined daily doses per 100 admissions (*P* = .006) and a 16% reduction per 1000 patient-days (*P* = .013). There was a 32% reduction in antimicrobial acquisition cost per admission (*P* = .013) and a 25% acquisition cost reduction per patient-day (*P* = .022).

**Conclusions:**

An effective antimicrobial stewardship program was implemented with limited resources on the medical-surgical service of a 100-bed community hospital.

## Background

Hospitals with less than 200 beds accounted for 72% of American Hospital Association-defined community hospitals in 2008 and 63% of the acute care facilities reporting to the National Healthcare Safety Network (NHSN) in 2010 [1,2]. Recent reports have demonstrated that large and small hospitals alike comparably share the problem of antimicrobial resistance. An analysis of data from the National Nosocomial Infection Surveillance (NNIS) System demonstrated parallel increases in antimicrobial resistance in *Staphylococcus aureus* blood stream infections, *Escherichia coli* urinary tract infections and *Pseudomonas aeruginosa* pneumonias within large and small acute-care facilities between the periods of 1990–1994 and 2000–2004 [3]. In addition, intensive care units of small and large hospitals reporting to NHSN were shown to have comparable proportions of device-associated infections with multidrug-resistant *Klebsiella pneumoniae* and *E. coli*[4]. Similarly, the proportion of *Acinetobacter baumannii* that was multidrug-resistant was comparable or greater in smaller facilities compared to larger, tertiary facilities. Furthermore, smaller bed size was independently associated with a higher rate of incident *Clostridium difficile* infection (CDI) cases reported among 210 Ohio acute care hospitals during 2006 [5].

Antimicrobial stewardship has been promoted for all hospitals to help cope with the challenges of CDI and emerging resistance to antibiotics [6-8]. In 2007, the Infectious Diseases Society of America (IDSA) and the Society for Healthcare Epidemiology of America (SHEA) published revised guidelines for developing institutional programs to enhance antimicrobial stewardship [9]. Several recent surveys have suggested antimicrobial stewardship programs (ASPs) may be more prevalent than appreciated from a review of the medical literature [10,11]. Nevertheless, a 2011 report of a survey of infectious diseases (ID) specialists of the IDSA Emerging Infections Network concludes that “small community hospitals still represent the ‘frontier’ for new stewardship programs” and that they are the hospitals “least likely to have ASPs, the least likely to provide compensation to physicians, and the least likely to believe that any outcomes data might convince administrators to support ASPs” [12]. Even so, smaller hospitals may have higher rates of antimicrobial use than those of large academic medical centers [13]. It has been nearly a decade since the only report of an ASP from a community hospital in the United States with less than 200 beds [14]. We report the implementation of a contemporary ASP on the medical-surgical service of a 100-bed community hospital.

## Methods

### Program setting

An ASP was implemented on the medical-surgical service of a full-service 100-bed community hospital located in an ethnically diverse community in metropolitan Dallas, TX. The 43-bed medical-surgical service consisted of a 24-bed medical-surgical floor unit, 11-bed progressive care unit, and 8-bed medical-surgical intensive care unit. There were no inpatient transplant services, specialized oncology, pediatric, psychiatric, or rehabilitation units. Medical subspecialties were broadly represented and included infectious diseases (ID); surgical specialties included cardiothoracic surgery, colorectal surgery, general surgery, neurosurgery and orthopedic surgery. An electronic medical record was available but without computerized physician order entry or electronic progress notes.

Throughout the reported period, a nurse staffed an infection prevention and control program that included surveillance and prevention activities for multidrug-resistant organisms, CDI, and device-associated infections. Since 2006 and throughout the reported period, an ID physician served as the medical director of infection control and employee health. Active admission surveillance testing for methicillin-resistant *Staphylococcus aureus* was performed on high-risk patients for isolation and cohorting purposes. An off-site laboratory performed microbiology services including preparation of an annual facility antibiogram and polymerase chain reaction testing for CDI. (Laboratory testing for CDI was performed on-site using an enzyme-linked immunoassay for *C. difficile* toxin prior to November 2009).

The pharmacy was staffed by 6.4 full-time-equivalent pharmacists, including a pharmacy director and clinical pharmacy supervisor. The pharmacists did not receive specialized training in infectious diseases.

### Interventions

During the baseline period (January 2009 – August 2009), the ID physician medical director of infection control, the clinical pharmacy supervisor and the pharmacy director together formed an Antimicrobial Stewardship Team (AST) and drafted an antimicrobial stewardship policy and program description for approval by the Pharmacy and Therapeutics Committee and Medical Executive Committee. AST members educated the medical staff about the program through presentations at medical staff committee meetings and through a continuing medical education conference. For approximately one hour twice weekly during the intervention period (September 2009 – December 2010), the ID physician and one or the other of the AST pharmacists audited medical records of inpatients on the medical-surgical service that were receiving more than two days of systemic antimicrobial therapy. On occasion, the AST audited other patient records with shorter durations of therapy. The AST members audited records for prescribed antimicrobial agent(s), clinical indication(s), planned treatment duration(s), drug allergies, renal function, pertinent laboratory and radiographic data. Non-binding written recommendations were made and placed in the record using a communication form that did not become part of the permanent medical record. There were no formulary restrictions or preauthorization requirements.

**Figure 1:**
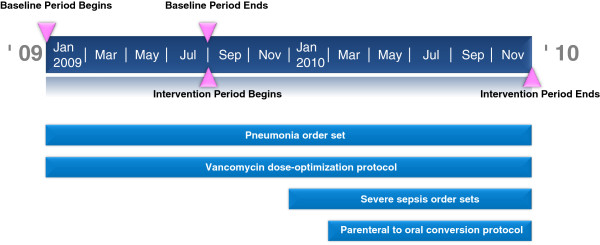
**Antimicrobial stewardship program implementation timeline.** The timeline depicts antimicrobial stewardship program milestones and interventions. A pneumonia order set and vancomycin dose-optimization protocol were implemented prior to the baseline period. Severe sepsis order sets and a parenteral to oral conversion protocol were implemented during the intervention period. The severe sepsis order sets included choices for initial antibiotic regimen by suspected source of infection and a schedule for continued therapy without automatic stop orders or requirements for physician justification. Individual agents included in the order sets were ampicillin, ampicillin-sulbactam, azithromycin, aztreonam, ceftriaxone, levofloxacin, linezolid, meropenem, piperacillin-tazobactam, rifampin and vancomycin. Antimicrobial agents in the parenteral to oral conversion protocol were fluconazole, levofloxacin, linezolid, metronidazole and voriconazole at the same dose and frequency.

Additional interventions implemented prior to the baseline period included automatic vancomycin dose-optimization and a pneumonia order set. Order sets for treating patients with severe sepsis or, suspected severe sepsis, and a parenteral to oral conversion protocol were implemented during the intervention period. A timeline with ASP milestones and interventions is displayed in Figure 1.

### Data collection

The AST categorized recommendations as follows: to discontinue all agent(s), to de-escalate antimicrobial therapy (to discontinue one or more agents and/or substitute an alternate agent(s) with decreased spectrum of activity), to limit duration, to broaden coverage (to add one or more agents and/or substitute alternate agents(s) with increased spectrum of activity), to optimize dose (e.g., for indication, renal function, and/or body weight), to convert route of administration from parenteral to oral and to consult ID. Pharmacist team members followed up recommendations and scored implementation. Data from record review and recommendations were tabulated monthly.

Admissions, patient-days (excluding outpatient-days), Medicare Case Mix Index, and characteristics of patients discharged from the medical-surgical service were obtained from hospital administrative databases. Facility-wide acquisition and/or return costs and quantities for all systemic antimicrobial agents (antibacterial, antifungal and antiviral) were recorded monthly from pharmacy records for the baseline and intervention periods. In addition, administered quantities of antimicrobials were recorded monthly for each patient care location of the medical-surgical service and defined daily doses (DDDs; World Health Organization Center for Drug Statistics Methodology) were calculated. Antimicrobial acquisition costs were estimated by multiplying administered antimicrobial unit quantities by the 2009–2010 facility average acquisition price per unit. Incident healthcare-facility-onset CDI cases were scored and incidence rates calculated per 10,000 patient-days according to the NHSN multidrug-resistant organism and CDI module protocol laboratory-identified event methodology [15].

### Statistical analysis

Patient characteristics, antimicrobial use and cost, and CDI rates were compared during baseline and intervention periods. The Mann–Whitney *U* test was used for continuous variable distributions, and the _*χ*_^2^ test was used for categorical variables. All reported *P* values were two-tailed with *P* less than .05 as the level of significance. Statistical calculations were performed using GraphPad Prism, version 5.04.

## Results

### Patient characteristics

There were 1,422 admissions and 5,572 patient-days on the medical-surgical service during the 8-month baseline period and 3,076 admissions and 11,109 patient-days during the 16-month intervention period. Patient characteristics of the medical-surgical service are summarized in Table 1. Primary diagnoses were similar between the two periods except for diseases of the circulatory system (*P* = .027). The mean monthly, facility-wide Medicare Case Mix Index increased from 1.4 to 1.6, or by 14% (*P* = .005).

**Table 1:** Patient characteristics during baseline and intervention periods of an antimicrobial stewardship program

**Variable**	**Baseline**	**Intervention**	***P***^**a**^
Discharges, no.	1409	3054	
Age, mean (SD), years	57.4 (18.6)	57.4 (18.7)	.933
Male sex	628 (44.6)	1304 (42.7)	.241
Race			
Asian	93 (6.6)	197 (6.5)	.850
Black	185 (13.1)	463 (15.2)	.074
Hispanic	180 (12.8)	377 (12.3)	.686
White	940 (66.7)	1971 (64.5)	.156
Health insurance			
Commercial, HMO, PPO	627 (44.5)	1274 (41.7)	.084
Medicare, Managed Care Medicare	639 (45.4)	1382 (45.3)	.951
Medicaid, Managed Care Medicaid	37 (2.6)	95 (3.1)	.374
ALOS, mean (SD), days	3.9 (0.3)	3.6 (0.3)	.118
Primary ICD-9 diagnosis code(s)			
Circulatory	314 (22.3)	593 (19.4)	.027
Diabetes mellitus	19 (1.3)	48 (1.6)	.569
Digestive	202 (14.3)	468 (15.3)	.391
Genitourinary	182 (12.9)	454 (14.9)	.083
Infectious and parasitic	57 (4.0)	110 (3.6)	.468
Musculoskeletal and connective tissue	61 (4.3)	160 (5.2)	.193
Neoplasms	144 (10.2)	293 (9.6)	.513
Respiratory	124 (8.8)	276 (9.0)	.797
Skin and subcutaneous tissue	43 (3.1)	81 (2.7)	.450

NOTE:

Data are no. (%) of discharged medical-surgical service patients, unless otherwise indicated.

^a^ - The Mann–Whitney *U* test was used to compare continuous data, and the χ^2^ test was used to compare categorical data.

Abbreviations: ALOS, average length of stay; HMO, health maintenance organization; ICD-9, International Classification of Diseases, 9^th^ Revision; PPO, preferred provider organization; SD, standard deviation.

There were 367 audits of 333 unique inpatient records during the stewardship period (Table 2). At the time of audit, 349 (95%) patients had been prescribed antimicrobial therapy for three or more consecutive days, 168 (46%) were receiving more than one agent, 206 (56%) were receiving fluoroquinolones (levofloxacin) and 13 (4%) were receiving two agents with anaerobic activity. Suspected or confirmed pulmonary infection was a rationale for antimicrobial therapy in 173 (47%) audits and the most common indication for prescribed antimicrobial therapy.

**Table 2:** Characteristics of antimicrobial prescription regimens from 367 audits of 333 unique patient medical records

**Variable**	**No. (%) of Audits**
Antimicrobial therapy > 2 inpatient days	349 (95.1)
Antimicrobial agent number	
1	199 (54.2)
>1	168 (45.8)
Parenteral agent(s) part of regimen	336 (91.6)
Fluoroquinolone^a^ part of regimen	206 (56.1)
Two anti-anaerobic agents^b^	13 (3.5)
Antimicrobial indications^c^	
Pulmonary	173 (47.1)
Urinary	90 (24.5)
Intra-abdominal	88 (24.0)
Skin and soft tissue	46 (12.5)
Other	25 (6.8)

NOTE:

^a^ - Levofloxacin was the only fluoroquinolone prescribed among the audited records.

^b^ - Amoxicillin-clavulanate, ampicillin-sulbactam, clindamycin, ertapenem, imipenem, meropenem, metronidazole and piperacillin-tazobactam.

^c^ - Patients were allowed more than one indication per record audit.

Characteristics of AST recommendations are summarized in Table 3. The AST advised no change to the prescribed antimicrobial regimen for 54 audits and made one or more recommendation(s) to change the prescribed antimicrobial regimen (or consult ID) for 313 audits during the stewardship period. Physicians implemented recommendation(s) from each of 234 (75%) audits, including from 85 of 115 for which discontinuation of all antimicrobial therapy was recommended.

**Table 3:** Characteristics of 313 AST audits with one or more recommendations

**Recommendation category**	**Number of audits**	**Implemented recommendations**	**Implementation rate (%)**
All	313	234	75
Discontinue all agent(s)	115	85	74
De-escalate^a^	65	53	82
Limit duration^b^	21	13	62
Consult infectious diseases	19	16	84
Optimize dose	14	7	50
Broaden ^c^	5	3	60
Convert parenteral to oral^d^	3	3	100
More than 1 category	71	54	76

NOTE:

^a^ - Discontinuation of one or more agent(s) and/or substitution of alternate agent(s) with decreased spectrum of activity.

^b^ - Limit duration for same agent(s) at same dose, route and schedule.

^c^ - Addition of one or more agent(s) and/or substitution of alternate agent(s) with increased spectrum of activity.

^d^ - Parenteral to oral conversion(s) of same agent(s) at same dose and schedule.

### Antimicrobial use and cost, and CDI

There was a 22% reduction in mean monthly use of all antimicrobial agents per 100 admissions (*P* = .006) and a 32% reduction in cost per admission (*P* = .013) in comparison to the baseline period (Table 4). Antimicrobial use and cost were also calculated per patient-day. There was a 16% reduction in mean monthly antimicrobial use per 1000 patient-days (*P* = .013) and a 25% reduction in cost per patient-day (*P* = .022). There were statistically significant reductions from baseline in the use of antipseudomonal carbapenems (imipenem and meropenem), clindamycin, levofloxacin, linezolid, trimethroprim-sulfamethoxazole, antibacterials and antifungals using either metric denominator; there was a statistically significant increase from baseline in the use of cefazolin.

**Table 4:** Antimicrobial use and cost during baseline and intervention periods of an antimicrobial stewardship program

**Variable**	**DDD per 100 Admissions**			**DDD per 1000 Patient-days**		
	**Baseline**	**Intervention**	***P***^**a**^	**Baseline**	**Intervention**	***P***^**a**^
By category						
Antibacterials	401.8	318.9	.009	1028	878.7	.011
Antifungals	23.1	13.2	.035	59.1	36.5	.047
Antivirals	7.5	5.2	.375	21.3	14.2	.257
All agents	432.4	337.3	.006	1109	929.4	.013
By selected class						
Antipseudomonal carbapenems^b^	13.8	4.4	.047	35.0	12.4	.047
Cephalosporins	42.2	49.2	.188	108.7	135.7	.030
Echinocandins	4.7	3.2	.975	11.3	8.7	.924
Fluoroquinolones	123.1	100.8	.011	314.2	279.2	.071
By selected agent						
Ampicillin-sulbactam	17.6	16.1	.830	46.8	43.9	.878
Cefazolin	20.8	26.9	.013	53.7	74.9	.004
Ceftriaxone	13.2	14.5	.603	34.3	39.8	.312
Cefepime	0.5	1.9	.217	1.3	5.0	.171
Clindamycin	22.5	12.7	.004	57.4	34.8	.009
Daptomycin	2.0	0.7	.913	5.1	2.1	.855
Ertapenem	14.2	12.4	.878	35.6	34.0	.830
Fluconazole	17.6	9.8	.105	46.1	27.2	.284
Levofloxacin	122.0	97.1	.006	311.2	269.2	.030
Linezolid	4.5	0.5	.017	11.3	1.6	.020
Metronidazole	21.7	16.9	.105	55.5	46.1	.257
Nafcillin	3.0	0.3	.370	7.3	0.9	.370
Piperacillin-tazobactam	32.0	30.8	.783	80.7	84.7	.736
Tigecycline	0.2	0.2	.702	0.5	0.4	.702
Trimethoprim-sulfamethoxazole	25.6	3.9	.016	65.0	10.8	.017
Vancomycin	44.8	42.7	.783	115.2	116.2	.976
	US$ per Admission			US$ per Patient-day		
	Baseline	Intervention	*P*^a^	Baseline	Intervention	*P*^a^
By category						
Antibacterials	79.8	54.7	.025	20.2	15.1	.040
All agents	87.0	59.4	.013	22.0	16.4	.022

NOTE:

Data are means of monthly medical-surgical service antimicrobial use and cost.

^a^ - The Mann–Whitney *U* test.

^b^ - Imipenem and meropenem (doripenem was not used). A formulary change (and auto-substitution policy) from meropenem to imipenem was implemented during the intervention period.

Abbreviations: DDD, defined daily doses (World Health Organization Center for Drug Statistics Methodology).

The mean monthly, incident healthcare-facility-onset CDI incidence rate for the medical-surgical service was 3.7 during the baseline period and 9.2 during the intervention period (*P* = .232).

## Discussion

In summary, we report the implementation of an ASP at a 100-bed community hospital employing a core strategy of post-prescriptive medical record audits and nonbinding AST recommendations with significant reductions in antimicrobial use and cost.

Respondents to a 2009 survey of the IDSA Emerging Infections Network on programmatic strategies and barriers for ASP implementation, reported that 61% of their hospitals had an ASP and that 12% were planning to initiate one [12]. However, respondents from hospitals with less than 200 beds reported that only 44% of their hospitals had an existing ASP; even so, the survey was thought to “likely overestimate the dissemination of ASPs”. Lack of funding and/or personnel were considered to be primary barriers to ASP implementation.

Additional challenges in the implementation of our program included data management and the creation of data management tools, program documents, procedures, and reports as well as educational materials for the medical staff.

Although other reports have been published about ASPs implemented in community hospitals [16,17], only one has previously described an ASP at a facility in the United States with less than 200 beds. In 2003, LaRocco described an antibiotic support team developed at a 120-bed facility in Louisiana [14]. Concurrent chart review was performed three days per week focusing on multiple, prolonged and high-cost antibiotic therapies. There was a 19% savings on antibiotic costs per patient-day over a 12-month intervention period. We employed a similar core strategy but with a 2-person ID physician/clinical pharmacist team that audited medical records two days per week. We limited our audits to patients on the medical-surgical service, as others have reported [18], and demonstrated a 25% cost reduction per patient-day (*P* = .022). LaRocco did not report use metrics; there may be other unknown differences in the setting, patient characteristics, interventions, and data analysis limiting any further comparisons between these two programs.

The generation of recommended antimicrobial use metrics was among our greatest challenges. We selected DDD as recommended by the IDSA/SHEA stewardship guidelines [9]. Days of therapy were not available from pharmacy records. These same stewardship guidelines are silent on the recommendation for a metric denominator(s); we calculated both DDD per admission and per patient day, as recommended by others [19-21].

There were few differences in patient characteristics between the baseline and intervention periods. The facility-wide Medicare Case Mix Index increased significantly during the intervention period; however it is unclear how this may be related to antimicrobial use on the medical-surgical service.

Severe sepsis order sets and a parenteral to oral conversion protocol were implemented during the intervention period. The order sets did not limit duration of antimicrobial therapy and the parenteral to oral conversion protocol included only five antimicrobial agents eligible for substitution at the same dose and frequency. Nevertheless, we cannot exclude the possibility that these additional interventions may have had an impact on antimicrobial use and cost.

Similar to other recent reports from the United States, we observed more than one antimicrobial agent was prescribed for 46% of audited records. In a retrospective study of adult, nonpsychiatric inpatients prescribed two or more consecutive days of antibiotic therapy at a tertiary care hospital in New York, two or more antibiotic agents were employed for 63% percent of 10,154 hospitalizations [22]. In an observational study of adult inpatients prescribed fluoroquinolones at a tertiary care hospital in Ohio, 56% of 227 regimens combined fluoroquinolones with antibiotic agents from other classes [23].

We observed a pulmonary source of infection was an indication for antimicrobial therapy in 47% of audited records. In addition, a fluoroquinolone, levofloxacin, was prescribed in 56% of audited records and accounted for approximately 30% of overall antibiotic consumption on the medical-surgical service during both the baseline and intervention periods. We speculate that these findings may in part be related to antibiotic choices in a pneumonia order set designed to align with the Centers for Medicaid and Medicare Services National Inpatient Quality Measure PN-6, “Initial Antibiotic Selection for Community-Acquired Pneumonia (CAP) in Immunocompetent Patients” [24].

Discontinuation of all antimicrobial therapy accounted for 36% (85/234) of audited records with implemented recommendations. The observed reductions in the use of levofloxacin, and of antimicrobials overall, would likely not have been realized if we had chosen a core strategy of antibiotic restriction focused on one or more high cost agent(s). Although inexpensive compared to other antimicrobials and available for oral administration, fluoroquinolones have been associated with both methicillin-resistant *S. aureus* and CDI in hospitals [25,26]. Receipt of fluoroquinolones and all antibiotics have also been shown to be independent risk factors for carbapenem-resistant *K. pneumoniae* acquisition among hospitalized adults [27].

Measurement of changes in antimicrobial resistance patterns associated with antimicrobial stewardship has been recommended as a potential outcome measure for ASPs [9]. In the current report, small numbers of unique clinical isolates precluded a meaningful assessment of the program’s impact on antibiotic resistance. Likewise, a switch from enzyme immunoassay detection of toxins to a polymerase chain reaction assay in month 3 (November 2009) of the 16-month ASP intervention period likely confounded comparison of healthcare-facility-onset CDI rates. (A recent report demonstrated significant increases and an approximate doubling of the prevalence of positive laboratory tests for CDI and the CDI rate after a similar switch in detection methods [28]). Also, factors other than antibiotic use may affect CDI, and we were unable to draw any conclusions about the statistically unchanged CDI rates between the periods [29].

## Conclusions

In conclusion, an effective ASP was implemented at a 100-bed community hospital. Importantly, there were significant reductions in overall antimicrobial use. More focus is needed on antimicrobial stewardship strategies, measures and resources in this healthcare setting.

## Competing interests

The authors declare that they have no competing interests.

## Authors’ contributions

DS, PP, AN, and FC shared in program conception and design. DS served as the physician champion for the medical staff. PP developed the data management tool and performed data analysis. DS, AN, and FC performed medical record audits. DS and PP drafted the initial manuscript. All authors have read and approved the final manuscript.

## Previous presentations

Presented in part: 21^st^ Annual Scientific Meeting of the Society for Healthcare Epidemiology of America, Dallas, TX, 1–4 April 2011 [Abstract 77].
